# Digital Intervention for Electronic Patient-Reported Outcomes in Advanced Cancer: Mixed Methods Study

**DOI:** 10.2196/91416

**Published:** 2026-06-18

**Authors:** Christina Sauer, Simeon Sauer, Elena Sophia Doll, Stefanie Zschäbitz, Jan Simon Raue, Maik Turni, Till Johannes Bugaj, Kiriaki Hiller, Imad Maatouk

**Affiliations:** 1 University Cancer Center Frankfurt (UCT), University Hospital Frankfurt, Goethe University, Theodor-Stern-Kai 7, Frankfurt, Hesse, 60590, Germany, 49 69-6301-83787, 49 69-6301-83788; 2 Technische Hochschule Mannheim, Mannheim, Baden-Wurttemberg, Germany; 3 Institute of Medical Psychology, University Hospital Heidelberg, Heidelberg, Baden-Wurttemberg, Germany; 4 Deptartment of Medical Oncology, University Hospital Heidelberg, Heidelberg, Baden-Wurttemberg, Germany; 5 Outcomes4Me Germany GmbH, Berlin, Berlin, Germany; 6 Department of General Internal Medicine, Psychosomatics and Psychotherapy, University Hospital Heidelberg, Heidelberg, Baden-Wurttemberg, Germany; 7 Department of Internal Medicine II, Division of Integrated Psychosomatic Medicine, Universitätsklinikum Würzburg, Wuerzburg, Bavaria, Germany

**Keywords:** cancer, electronic patient-reported outcome, ePRO, digital intervention, eHealth, immunotherapy, immune-checkpoint inhibition

## Abstract

**Background:**

Digital health interventions are increasingly being integrated into oncological care to support patients in managing treatment-related symptoms and psychological distress. In a randomized controlled pilot trial, we investigated the feasibility and preliminary efficacy of a digital health app (SOFIA) among patients with cancer, including those in palliative care. SOFIA consists of an electronic patient-reported outcome (ePRO) assessment and coaching component. We showed good feasibility and high acceptability of SOFIA in routine clinical care.

**Objective:**

This pilot study aimed to evaluate user experiences and behavior with SOFIA. We applied a mixed methods design, combining a qualitative exploration of patients’ experiences and a quantitative analysis of app usage patterns. The integrated findings are intended to inform the refinement and further development of digital health interventions to better address the needs, preferences, and engagement behaviors of this patient population.

**Methods:**

Patients randomly assigned to the intervention group participated in semistructured interviews at 2 time points during the 12-week intervention period: midway through the intervention (at week 6) (T1) and postintervention (at week 12) (T2). Qualitative data were analyzed using content analysis. User data were descriptively analyzed with Python and the Pandas library (version 2.2.3).

**Results:**

Our qualitative analysis of 29 patients revealed benefits regarding the ePRO assessment (empowerment, support, user-friendliness, and facilitation of patient-physician communication), as well as some criticism (inflexible design and insufficient use by physicians). Benefits of the coaching tool included the availability of helpful information and design aspects such as clarity and user-friendliness. Quantitative data from 32 active users of the app showed that 16 (50%) of patients read articles, 28 (87.5%) started journeys, and 15 (46.9%) completed exercises at any point during the study.

**Conclusions:**

The results of this mixed methods study may provide important indications for digital interventions, including ePRO assessment, for patients with cancer. From the patients’ perspective, key features include intuitive design, relevant symptom items, reliable reminder functions, the translation of ePROs into clinically actionable information, clear symptom visualizations, seamless integration into electronic health records, and effective physician engagement. Both our qualitative and quantitative data show the importance of therapy-specific content. These results might increase acceptance and usage, and thereby also the clinical benefit, of future digital interventions for patients with cancer.

## Introduction

Digital health interventions (DHIs) and the recording of electronic patient-reported outcomes (ePROs) have become an important part of psycho-oncological care. The systematic collection of ePROs has demonstrated substantial benefits in oncology, enhancing communication, quality of care, and clinical efficiency. ePROs foster more effective dialogue between patients and healthcare professionals by providing structured, continuous insights into patients’ symptoms and well-being [1]. This facilitates more open, targeted, and meaningful communication during clinical encounters [2]. Evidence further indicates that integrating ePROs into oncology care leads to improved outcomes, including higher health-related quality of life (HRQoL) [3], more effective symptom management, and even prolonged survival among patients with cancer [4,5]. The use of ePROs supports a patient-centered approach by enabling treatment decisions that are more closely tailored to the individual needs, preferences, and experiences of patients. Moreover, ePRO systems allow for the early detection of clinical deterioration and deterioration of HRQoL [6]. Finally, regular monitoring enables timely interventions that can prevent complications and reduce emergency room visits [7].

DHIs are increasingly being integrated into oncological care to support patients in managing treatment-related symptoms and psychological distress. Previous qualitative research with patients with breast cancer has shown that such interventions are perceived as acceptable and hold strong potential to reduce anxiety, address gaps in care, and provide reassurance throughout the treatment process [8]. Similarly, in the study by Krakowczyk et al [9], patients with various cancer entities evaluated the usefulness and usability of *Make It Training*, a modular digital mental health intervention designed to reduce psychological distress and improve coping and HRQoL. Patients described the intervention as user-friendly and supportive in developing functional coping strategies, highlighting its potential for integration into daily life and routine oncological care. However, to date, evidence on the acceptability and perceived usefulness of such DHIs [10] among palliative patients remains scarce, although this group faces particularly severe physical and psychological challenges due to the advanced stage of their illness.

In the SOFIA pilot trial, we developed and tested a mobile app for patients undergoing immunotherapy with immune checkpoint inhibitors (ICIs) within a randomized controlled design. The SOFIA app comprised 2 components: *SOFIA Monitoring*, which involved the regular assessment of ePROs, and *SOFIA Coaching*, which provided tailored information related to treatment and illness management. In a previous publication, we demonstrated the high feasibility and acceptance of the app in clinical routine, with an adherence rate of 91% for completing ePRO assessments twice weekly over a 3-month intervention period. Patients in the intervention group (IG) reported significantly lower levels of depression and distress, as well as improved HRQoL, compared to those receiving treatment as usual [11].

Several studies have qualitatively investigated expectations and experiences with DHIs in patients with cancer. In a recent meta-analysis, Zhang et al [12] synthesized 12 qualitative studies on user experiences among older patients with cancer, identifying 3 overarching themes: (1) positive experiences, (2) barriers to use, and (3) user expectations. The included studies varied in terms of app content (eg, ePRO assessment, physiological tracking, or combined DHIs such as ONKOkompass) and also addressed expectations and needs. The review provides an important overview of key themes and topics. However, to compare specific benefits and barriers, it is necessary to investigate these aspects separately for each component (ePRO assessment, coaching tools) in patients who have actually used the app.

Similarly, a recent systematic review by Lai-Kwon et al [10] reported that ePRO-based systems for monitoring immune-related adverse events are generally feasible and acceptable in patients receiving immunotherapy. However, the included studies predominantly focused on symptom monitoring, with limited or no systematic evaluation of additional intervention components such as coaching modules.

Evidence on the usefulness of ePRO-based and combined eHealth interventions in patients receiving ICI therapy remains limited. Tolstrup et al [13] investigated the usefulness of an ePRO assessment in 14 patients with melanoma receiving ICI therapy and found that patients perceived the tool as easy to access and meaningful to complete. In addition, Glaser et al [14] evaluated the CAPABLE system, an eHealth intervention including symptom management and coaching features in patients undergoing ICI therapy. Their mixed methods study reported high overall perceived usefulness, with more than 75% of participants rating the system positively. However, the evaluation was conducted at the system level and did not distinguish between experiences with the ePRO and coaching components.

Overall, to date, no study has included a mixed sample of patients with cancer undergoing ICI therapy and quantitatively investigated their experiences with ePRO assessment and coaching components separately. Furthermore, no study has combined qualitative data with user log data, which provide important insights into actual usage behavior.

This study aimed to investigate acceptability and patient engagement with the app, operationalized as (1) qualitative patient perspectives on using the SOFIA app, assessed separately for the ePRO assessment and coaching components, and (2) quantitative usage behavior. These insights are intended to inform the future development and optimization of DHIs for patients with cancer, including those in palliative care and undergoing ICI therapy.

## Methods

### Study Design

This study was embedded within a pilot study and used a convergent parallel mixed methods approach [15], combining (1) qualitative exploration of patients’ experiences of app use and (2) quantitative analysis of user behavior. The underlying phenomenon of interest was patient experience with both components of the SOFIA app, conceptualized as the interplay between patients’ subjective experiences and their observable usage behavior. The mixed methods design was chosen to capture both dimensions and to better understand the mechanisms shaping engagement.

The study was a prospective single-center, 2-arm randomized controlled external pilot trial. The study protocol and details of the study methods are described elsewhere [11,16].

To estimate the key parameters for the main trial, a total of 70 participants (35 per group) were required, based on the simulation-based approach for determining sample size in external pilot trials with continuous outcome variables [17].

### Intervention

Participants in the IG received access to the SOFIA app (for a detailed description, [11]). SOFIA was integrated into the Mika app, a digital therapeutic providing individualized supportive care and aiming to reduce psychological distress in people with cancer. The app entails different components, including symptom monitoring and coaching modules (for a detailed description, [11]).

Monitoring: SOFIA Monitoring comprised 11 physical and up to 9 mental ePROs, assessed twice weekly with email reminders. Physical symptoms were selected by experienced medical oncologists based on European Society for Medical Oncology guidelines [18] and included weakness, diarrhea, melena, dry cough, dyspnea, reduced urinary output, joint and muscle pain, skin toxicity, fever, and jaundice.

Coaching: The coaching component included modules such as Discover and Journeys. The module “Discover” is the content library of the Mika app, consisting of articles and videos with easily understandable and evidence-based information on several cancer-related topics (eg, cancer and treatment types, nutrition in cancer; Table S1 in Multimedia Appendix 1), written and peer-reviewed by medical experts. An integrated statistical content recommendation engine delivers individual content recommendations to the user based on age, gender, cancer type, therapy type, and reported symptoms. By considering the symptoms the patient has reported in the symptom monitoring module, as well as patterns in their reading behavior, the algorithm predicts the likelihood of the user engaging with specific content and, based on the estimated read probability, presents the respective content to the user.

“Journeys” are the resource-activating psycho-oncological training courses of the Mika app that aim to help patients cope with cancer-associated distress in different areas (eg, living with immunotherapy; Table S1 in Multimedia Appendix 1). There are 13 journeys, each of which entails 3 subsequent stages, which in turn include 3 exercises. Exercises can be allocated to 1 of 4 categories: knowledge, mindfulness, relaxation, or physical exercise.

### Study Procedures and Analyses

#### Qualitative Approach

Reporting of the qualitative part adheres to the COREQ (Consolidated Criteria for Reporting Qualitative Research) [19] (Checklist 1). Patients in the IG participated in semistructured interviews at 2 time points during the 12-week intervention period: midway through (at week 6) (T1) and postintervention (T2). The interview guide is available in Multimedia Appendix 2. Questions were developed based on expert discussions and experiences from previous studies, with the aim of gathering information on experienced benefits and suggestions for improving SOFIA Monitoring and Coaching. The 1:1 interviews were conducted in German by 3 female study assistants who were master’s-level psychology students. Owing to their psychology background, all had well-developed communication skills and received additional briefing and supervision from the study coordinator (CS) prior to data collection. To minimize the burden on participants, the interviews were conducted by telephone. Some participants were already acquainted with their individual interviewer through the recruitment contact. At the beginning of each interview, the study assistants introduced themselves as study assistants in the SOFIA project; no other interviewer characteristics were shared. The interviews lasted about 10 minutes, depending on how detailed the answers were. Due to the brevity of the questionnaire and the limited selection of questions, no pilot testing or audio recording was conducted. The study assistants wrote down the statements by hand (either verbatim or summarized in bullet points) and then transferred them anonymized into an Excel spreadsheet. Additional field notes were not made. Interview notes were not returned to participants for comment and/or correction.

Statements were analyzed using structured qualitative content analysis, combining deductive and inductive approaches [20]. While the interview guide provided an initial set of categories (deductive), these categories were refined inductively based on the participants’ responses, with subcategories and hierarchical levels being developed during the course of the analysis. Initially, meaningful content aspects within each statement were identified as coding units and assigned to the overarching categories from the interview guide by 2 independent raters. As individual statements could contain multiple content aspects, more than 1 category assignment per statement was possible. The allocation was subsequently discussed within the research team. Disagreements were discussed until consensus was reached. In cases where differing opinions persisted, the senior researcher and study coordinator (CS) made the final decision. This procedure for resolving disagreements was applied consistently across all stages of the coding process. In the next step, common groups (subcategories) were formed within the overarching categories based on the assigned content aspects. Where appropriate, these subcategories were further consolidated into generic categories through additional team discussions. The final category structure emerged through an iterative, consensus-based coding process involving multiple rounds of analysis and regular team discussions. During this process, categories and subcategories were continuously reviewed, refined, and reorganized where substantial overlap was identified. In our overview, we have summarized all statements that were mentioned more than twice.

#### Quantitative Approach

SOFIA app usage was analyzed using log data of patients’ in-app activities, providing detailed insights into the types of content and exercises with which patients were most engaged. After participants in the IG received a study access code and consented to the app’s privacy terms, their pseudonymized in-app activities were automatically recorded, allowing the evaluation of engagement with different coaching features of the app. For the current analyses, the app modules “Discover” and “Journeys” were of interest.

The raw log files were preprocessed by the app provider (Fosanis GmbH), resulting in several spreadsheets containing general user data and logs of articles read in the “Discover” module, as well as logs of journeys started or completed and exercises performed. The spreadsheets included the following information: unique user ID; name of the article, journey, or exercise with which the user was engaged; period during which the engagement took place (weeks 0‐12: intervention phase; weeks 12+: follow-up phase); and the duration (in seconds) of the engagement.

The spreadsheets were then processed further using a customized Python script and the Pandas library (version 2.2.3) for data processing. Sessions of zero duration were removed, and the time unit was converted from seconds to hours. Finally, the descriptive statistics of user engagement presented in the *Results* section were produced.

### Ethical Considerations

The Ethics Committee of Heidelberg University Hospital (reference S-581/2018) approved the trial protocol, and the study was registered in the German Clinical Trial Register (reference DRKS00021064). All participants were informed about the study procedures and signed informed consent before being included in the study.

## Results

### Participant Characteristics

Of the 34 patients in the IG, 2 patients left the study directly after randomization. For the remaining 32 participants, user data were available. Regarding the availability of interview data, 29 patients participated in the midway assessment (T1), while 23 patients participated in the postintervention assessment (T2). One patient discontinued participation due to dissatisfaction with the application, 2 patients died during the study period, 1 patient was physically unable to participate in the interview, and 2 patients could not be reached by phone.

Patient characteristics are presented in Table 1. The study flow chart can be found in Multimedia Appendix 3. For a detailed explanation of the study flow [11].

**Table 1.** Patient characteristics (n=29).

Characteristics	Values
Age (y), mean (SD)	61.2 (11.4)
Time since diagnosis (mo), mean (SD)	25.3 (42.6)
Male sex, n (%)	19 (65.5)
Tumor entity, n (%)	
Urological	11 (37.9)
Gastrointestinal	7 (24.1)
Hepatobillary	5 (17.2)
Head and neck	1 (3.4)
Lung	1 (3.4)
Gynecological	2 (6.9)
Squamous cell, not other specified	2 (6.9)
Metastasis (yes), n (%)	27 (93.1)
ECOG^a^, n (%)	
0	13 (44.8)
1	16 (55.2)
Immunotherapy type of regimen, n (%)	
ICI^b^ (monotherapy)	13 (44.8)
ICI + ICI	8 (27.6)
ICI + TKI^c^	6 (20.7)
ICI + CHX^d^	2 (6.9)
Previous cancer treatments (yes), n (%)	25 (86.2)
Previous treatments, n (%)	
Radiation	2 (6.9)
Surgery	6 (20.7)
Chemotherapy	3 (10.3)
Immunotherapy	5 (17.2)
>1 previous cancer treatments	16 (55.2)
Psychiatric side diagnosis, n (%)	1 (3.4)

a ECOG: Eastern Cooperative Oncology Group Performance Status

b ICI: immune checkpoint inhibitor.

c TKI: tyrosine kinase inhibitor.

d CHX: chemotherapy.

In our IG sample, 6 patients received steroids due to adverse events after the start of immunotherapy. Five patients had brain metastases, resulting in 1 patient undergoing surgery, 3 receiving radiotherapy, and 1 death. No patients had meningeal metastases. Five patients had thyroid dysfunction: 3 alone and 2 combined with other side effects.

### Qualitative Results

**Figure 1.**
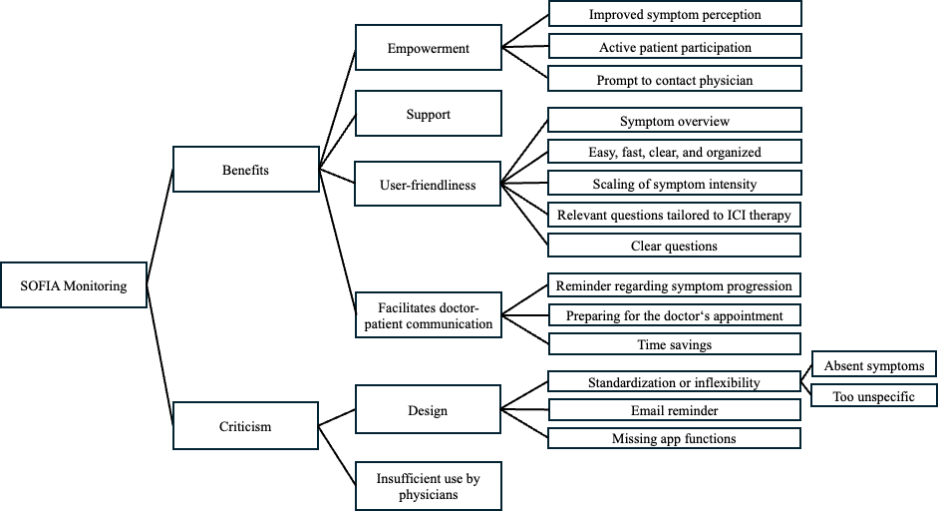
Main categories, generic categories, and subthemes of SOFIA Monitoring. ICI: immune checkpoint inhibitor.

For both SOFIA Monitoring and SOFIA Coaching, we identified two main categories: (1) perceived benefits and (2) criticism or recommendations. In the following, we describe the corresponding generic categories and subcategories for each and present illustrative quotations from the interview notes. The quotations were translated into English by CS. The numbering is based on the order in which the statements (eg, #1) and their documentation appear in the Excel spreadsheet (eg, *a*=first data spreadsheet), as well as the measurement time (T1 vs T2). Figures1  2 summarize the results of the qualitative analysis by presenting the coding structure with main categories and subcategories.

**Figure 2.**
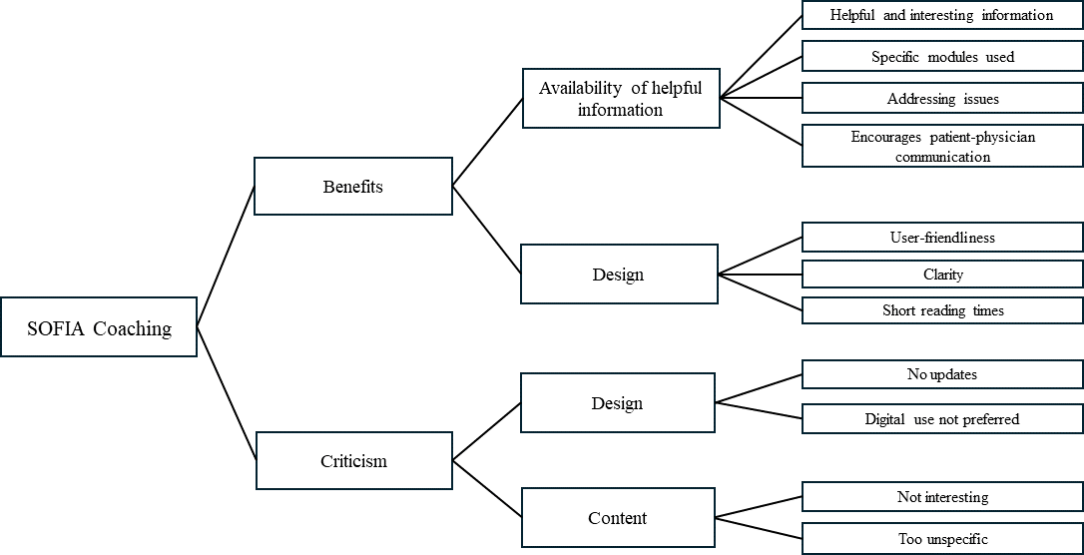
Main categories, generic categories, and subthemes of SOFIA Coaching.

### SOFIA Monitoring

#### Main Category 1: Benefits of SOFIA Monitoring (Assessment of ePROs)

##### Generic Category: Empowerment

Patients reported at T1 and T2 that the ePRO assessment improved their symptom perception (subcategory: improved symptom perception), strengthened their patient participation (subcategory: active patient participation), and the prompt to contact the physician (subcategory: prompt to contact the physician) encouraged them to become active or more aware of severe symptoms. These subcategories were combined to create the generic category called “empowerment,” since empowerment is the process by which patients gain the knowledge, skills, confidence, and control necessary to make informed decisions about their health and to play an active role in their own care [21].

###### Subcategory: Improved Symptom Perception

Patients reported that the ePRO assessment actively encouraged them to recognize their symptoms, including psychological ones, and that this helped them classify the symptoms:

*This is very helpful for assessing yourself better. Example: When you subjectively feel unwell and then objectively evaluate that feeling in the app*.

[#1a, T1]

*Also good for recording changes [was it just a one-off?]. If you just wrote it down, you wouldn’t have such a clear overview*.

[#3a, T1]

*You become aware of the questions and symptoms. Otherwise, I would not concern myself with them, but rather suppress them, especially when they concern depression*.

[#29a, T1]

*This helped me realize that I am doing well*.

[#1a, T2]

*Paying more attention to side effects [‘listening to my body more closely,’ ‘checking more often for certain symptoms,’ being more attentive to my body]*.

[#38a, T2]

*Deciding whether to go to the doctor or not*.

[#40a, T2]

###### Subcategory: Active Patient Participation

Patients reported that the ePRO assessment enabled them to play an active role in managing their condition:

*Being actively involved in documenting one’s own recovery and being able to observe progress and see where the journey is headed*.

[#25a, T1]

*Being part of something bigger and, therefore, being able to help future patients*.

[#26a, T1]

###### Subcategory: Prompt to Contact the Physician

Patients found the warning notice, along with the contact details, helpful in determining when to contact a physician:

*[...] A notification to contact a doctor, because not everyone does that [‘there’s time/it’s not urgent’], but when the notification keeps appearing, you decide to take action*.

[#4a, T1]

*You can see when something is above range and are informed to contact your doctor in that case, or that everything is OK*.

[#45a, T1]

### Generic Category: Support

Patients reported that they had the feeling that somebody was interested in their symptoms and that they were not alone with their fears. The patients felt reassured knowing what the typical symptoms were, which gave them a sense of security.

*I don’t feel left alone with my fears; I feel like someone cares about me*.

[#52a, T1]

*It’s good that someone is asking about my symptoms twice a week, not just every 3 weeks*.

[#2a, T2]

### Generic Category: User-Friendly Application

The patients appreciated the clarity of the presentation, the scaling of symptom intensity, and the relevant questions tailored to immunotherapy. They found the application easy and quick to use. See Table 2 for subcategories and examples.

**Table 2.** Subcategories of user-friendliness.

Subcategory	Statement (example)
Symptom overview	#5a (T1): You can view the scale yourself over a period of 30 days to get a good overview.
Easy, fast, clear, and organized handling	#42a (T2): Questions clear, given answer options good. #10a (T1): […] fast, clear questions.
Scaling of symptom intensity	#10a (T1): The range of 1‐10, because it can fluctuate daily. #23a (T1): The alignment with immunotherapy is satisfactory.
Relevant questions for ICI-therapy^a^	#12a (T1): You don’t have to figure out what is relevant yourself.
Clear questions	#73-74a (T2): Very easy, works well. #43a (T2): The questions are clearly formulated, and the predefined response options are well constructed.

a ICI: immune checkpoint inhibitor.

### Generic Category: Facilitation of Patient-Physician Communication

Some patients reported that the ePRO helped them remember symptoms for the physician-patient consultation and to report them during the consultation (subcategory: reminder regarding symptom course). Patients also used it to prepare for consultations with their physicians (subcategory: preparation of questions for the medical consultation). They also saw it as a time saver, as it meant that physicians were already able to obtain a detailed overview, facilitating communication and the identification of relevant symptoms (subcategory: time savings). Examples are depicted in Table 3.

**Table 3.** Subcategories of facilitation of patient-physician communication.

Subcategory	Statement (example)
Reminder regarding symptom course	#1c (T2): Yes, in the sense that you don't forget anything and have accurate information (even if something only happened once, which you would otherwise have forgotten to mention).
Preparation of questions for the medical consultation	#10c (T1): Yes, because you are more aware of what happened and what you want to say.
Time savings	#3c (T2): Yes, because the doctor already had my medical history, I didn’t have to explain everything again and could focus on what was important.

### Main Category 2: Criticism or Recommendations of SOFIA Monitoring

#### Generic Category: Design

##### Subcategory: Standardization or Inflexibility

The main criticism from patients concerned the standardization of the ePRO assessment in the app. Some patients reported missing symptoms (eg, hypertension or hypotension, weight gain/loss) and expressed a desire for greater specificity of items or a free text field for entering symptoms.

*Option to enter an individual symptom*.

[#2e, T1]

*Precise definition, what kind of pain, more specific*.

[#26e, T1]

*Difficult ‘in recent days’ because I always have muscle pain [unrelated to cancer]*.

[#29d, T1]

##### Subcategory: Email Reminder

Patients reported that they were unsatisfied with the email reminder function. They missed a direct reminder of the ePRO assessment via the app because, in some cases, there was no connection between the email reminder and the app (eg, #21d(T2): “Difficulties remembering to enter Mondays and Thursdays (as there is no link between mobile phone), did not make good use of the reminder function in the app.”).

##### Subcategory: Missing App-Function

Some patients reported that they missed some functions in the SOFIA Monitoring component, for example,

*You cannot enter anything if there are changes in the therapy*.

[#13f, T1]

*Doctors should be more proactive in approaching patients; if abnormal values are detected, it should not be up to the patient [who is already under stress] to report them*.

[#15f, T1]

### Generic Category: No Usage of Physicians

At T1, 62% (18/29) of patients reported that physicians did not use their input during the consultation; 17% (5/29) indicated that physicians used it partially, and 7% (2/29) reported that their input was used; 14% (4/29) were unable to judge that. At T2 (n*=*21 answers were available for this question), 14 (67%) patients reported no use of their input, 3 (14%) reported partial use, and 4 (19%) reported use. Patients expressed a desire for more feedback and a better integration of the ePRO assessment into their treatment.

### SOFIA Coaching

#### Main Category 3: Benefits of SOFIA Coaching

##### Generic Category: Availability of Helpful Information

Patients appreciated the availability of helpful and interesting information about their illness and treatment (subcategory: helpful and interesting information), as well as specific modules such as thematic journeys, the contact module, and a video interview (with an immunotherapy expert from the National Center for Tumor Diseases Heidelberg) (subcategory: specific modules used). Patients also valued being made aware of relevant issues (subcategory: addressing issues), which in turn facilitated communication with their physician (subcategory: encourages patient-physician communication). Examples are depicted in Table 4.

**Table 4.** Subcategories of availability of helpful information.

Subcategory	Statement (example)
Helpful and interesting information	#2f (T1): One can glean useful tips from it. #7f (T1): I liked it. Explanation of immunotherapy, themed trips, finding your way to cope with cancer-Positive: Content seemed very diverse, eg, something for all age groups (children/pensioners)
Specific modules used	#33f (T1): Thematic journeys, approximately every 2 days for half an hour to an hour, are helpful. #13f (T1): Video interview was good. #3f (T1): It’s good to know who to turn to (contact persons just a click away), in good hands. (contact module)
Addressing issues	#1f (T1): It’s good that it’s included in the app, because otherwise you might not bother with it. Depending on what’s happening that day, you can take another look at it
Encourages patient-physician communication	#40f (T1): I looked at thematic journeys, which raised questions that I then asked during my consultation with the doctor

##### Generic Category: Design

We identified 3 subcategories (user-friendliness, clarity, and short reading times) in the Design category. Patients appreciated the user-friendliness of SOFIA Coaching. They highlighted its ease of use, clarity, and short reading time (eg, #15f (T1): “The reading time is very helpful because my ability to concentrate is limited by my illness”).

### Main Category 4: Criticism or Recommendations for SOFIA Coaching

#### Generic Category: Design

Patients criticized missing updates (subcategory: missing updates). Some patients reported they generally preferred an analog information (subcategory: digital use not preferred). Examples are depicted in Table 5.

**Table 5.** Generic categories of improvable design aspects.

Subcategory	Statement (example)
Missing updates	#13f (T1): There are no updates/news that would encourage one to revisit the site. #15f (T1) Push notifications for new (would be helpful)
Digital use not preferred	#21g (T1): Does not use Coaching because he does not spend much time in front of the screen and is not a “computer freak.” #45g (T2): Technical side: He has to print out anything he finds interesting. There is no offline/paper function for marking things or making notes. He would prefer a kind of “wiki“ to better manage the information (information on study results). (…)

#### Generic Category: Content Not Interesting or Unspecific

Few patients reported they experienced the content and wished partly more detailed information about specific topics (eg, cancer and genetics).

*Individual to each person; article very general, much of it did not apply*.

[#52g, T2]

*I would like more information about the specific type of cancer*.

[#44f, T2)]

### Quantitative Results

Based on the log data of the 32 active users of the coaching features of the SOFIA app, we found that 16 (50%) of them had read at least 1 article from the “Discover” module. The median number of articles read per user in this group was 7.5. The corresponding mean value of 14.8 (SD 19.9) was distorted by 2 outliers (52 and 72 articles). As seen from the more detailed analysis in Table 6, more articles were read in the intervention period of the study than in the follow-up period.

**Table 6.** Analysis of articles read per user in the “Discover“ module of the SOFIA app (N=32).

Time period	Users with ≥1 articles read, n (%)	Number of articles read in this group, median (IQR)	Number of articles read in this group, mean (SD)
Any period	16 (50)	7.5 (12.5)	14.8 (19.9)
Intervention period only	16 (50)	4 (10)	10.6 (14.6)
Follow-up period only	7 (21.9)	4 (9)	9.6 (11.6)

The app organizes articles in different categories. The most frequently read category was “cancer therapy” (*k*=47), closely followed by “COVID and cancer” (*k*=38), “relieving symptoms” (*k*=37), “healthy lifestyle” (*k*=33), and “nutrition and cancer” (*k*=28). All data are depicted in figures in Multimedia Appendix 4 (pie chart, bar chart, and box plot).

The log data of the “Journeys” module of the SOFIA app revealed that 87.5% (28/32) users started at least 1 journey; 25% (8/32) of them completed at least 1 journey. Table 7 shows that most of the engagement in journeys took place in the intervention period.

**Table 7.** Analysis of journeys started or completed in the “Journeys“ module of the SOFIA app (N=32).

Time period	Users with ≥1 journey started, n (%)	Number of journeys started in this group, median (IQR)/mean (SD)	Users with ≥1 journey completed, n (%)	Number of journeys completed in this group, median (IQR)/mean (SD)
Any period	28 (87.5)	1 (1.25)/1.96 (1.62)	8 (25)	1 (2.00)/1.75 (1.04)
Intervention period only	26 (81.3)	1 (1.75)/1.96 (1.46)	6 (18.8)	1.5 (1.75)/1.83 (0.98)
Follow-up period only	4 (12.5)	1 (0.00)/1.00 (0.00)	3 (9.4)	1 (0.00)/1.00 (0.00)

Table 8 shows the popularity of journeys among users. By far, the most popular journey was “Living with immuno-therapy.” Figures in Multimedia Appendix 5 show the titles and frequencies of journeys completed.

**Table 8.** Frequency of journeys started.

	Users, n
Living with immunotherapy	24
Find your way	8
Relieve exhaustion	6
Gain control	5
Activate power source	4
Reduce stress	4
Nutrition and cancer	2
Managing emotions	1
Yoga and cancer	1

In the SOFIA app, journeys include exercises. Fifteen out of 32 (46.9%) users were engaged in at least 1 such exercise. In this group, an average of 9.1 (SD 8.8) exercises were started (median 9, IQR 6.5). Figures in Multimedia Appendix 6 show the most frequently started exercises. Multimedia Appendix 7 includes a joint display table linking the main qualitative themes of SOFIA Coaching to their respective quantitative usage patterns.

## Discussion

### Principal Results

DHIs are an integral part of oncological care. The evidence for this is very clear. Many efficacy studies do not provide accurate user information. The results of our convergent parallel mixed methods approach showed that, qualitatively, patients reported feelings of empowerment and support, appreciated the user-friendly design, and reported perceived improvement of patient-physician communication (ePRO assessment) as well as access to helpful and validated information (coaching component). Simultaneously, they wished for more flexibility in the ePRO assessments and more integration of the ePRO assessment into the clinical routine, as well as more specific data. Regarding the coaching component, some participants wished for more updates or did not prefer digital use. Quantitative user data indicated that over 50% (16/32) of patients read articles, engaged with journeys, and performed exercises, with immunotherapy-relevant articles, the management of side effects, and living with immunotherapy being of particular interest.

### Implications for ePRO Assessments

The results of our main study revealed high acceptability and feasibility of the ePRO assessment in patients undergoing ICI therapy, with an adherence rate of 91% [11]. We conducted qualitative interviews to gain deeper insights into user perspectives and needs. The findings of our accompanying qualitative study corroborate those of a previous investigation involving 186 patients with various cancer entities undergoing diverse cancer therapies, as well as 55 clinicians [22]. That study examined patient and clinician perspectives on the use of a DHI for collecting ePROs. Three overarching themes were identified for both stakeholder groups: (1) acceptability and functionality (relevance of symptom items, usability, reminders, perceived health status); (2) impact on clinical care (information and resource provision, contribution to research, self-monitoring, reassurance, connection to the hospital, and support for guided decision-making); (3) personal value of using eRAPID—the tool used for ePRO assessment (clinician engagement with eRAPID, facilitation of consultations, and support for medication or treatment adjustments) [22]. Furthermore, the results are in line with the recently published systematic review of qualitative data [12] identifying “improved self-management,” “safe and accessible support,” and “better access to health information” as key benefits, and “inadequate content design,” “technical difficulties,” and “negative user perceptions and behaviors” as key barriers. Consistent with our generic categories—user-friendly application (acceptability and functionality), facilitation of patient-physician communication (impact on clinical care), and empowerment, support, or availability of helpful and interesting information (personal value)—these findings provide several implications for the development of future DHIs including ePRO assessments in oncology. First, ePRO systems should prioritize high acceptability and usability by ensuring intuitive design, relevant symptom items, and reliable reminder functions (eg, directly via the app) [2]. Second, features that translate PROs into clinically actionable information—such as automated alerts or structured summaries—are essential to support timely decision-making. Third, future tools should actively facilitate patient-physician communication through clear symptom visualizations and seamless integration into electronic health records. In this context, ePROs could be particularly useful for gathering information about how patients tolerate therapies such as tyrosine kinase inhibitors. These therapies are often used in combination with ICIs and are frequently associated with chronic side effects that are difficult to assess during routine consultations, but which can have a significant impact on HRQoL [23].

Moreover, ePRO systems should promote patient empowerment by enabling self-monitoring, offering feedback on symptom trajectories, and providing access to supportive resources. Successful implementation further requires strong clinician engagement and incorporation into routine workflows. The results of our previously published study [11] revealed that 65% (n=28) of physicians (who treated patients in the IG) never or seldom used the graph for monitoring patients’ care—even though over 50% of physicians evaluated ePROs as useful and saw significant benefits in managing treatment with ICI (eg, in preparing for consultations or detecting side effects). These reports are in line with the results of the answers of our patients, as over 60% of patients reported no usage of their ePRO assessments in the clinical routine, but patients wished that their reports would be better integrated into the patient-physician consultation. It is important to note that these implications are derived from qualitative perceptions and descriptive usage patterns and should not be interpreted as evidence of causal mechanisms at an individual level.

In future studies, the integration of ePRO data into clinical workflows should be systematically evaluated, including the role of physician training. In line with studies showing that the assessment of ePROs and screenings should have consequences and aftercare [24], this is important for patients’ adherence to reporting ePROs regularly. It would then be particularly interesting to gain a deeper understanding of patients’ and physicians’ perspectives when ePROs are more integrated into clinical encounters.

Finally, offering flexible assessments while simultaneously enabling standardized data capture for research and quality improvement will be crucial for maximizing the clinical utility of next-generation ePRO solutions.

Furthermore, the factor of personal value (empowerment, support, availability of helpful information) might have led to improved HRQoL and reduced psychological burden in patients in the IG compared with the control group in our sample [11]. However, this interpretation should be viewed with caution, as our small sample size did not allow us to demonstrate the mediating effects of these factors.

### Implication of Coaching Components and Integration of Quantitative Findings

Patients reported benefiting from the availability of relevant and helpful information, as well as from the user-friendly and clear design. This is consistent with previous studies showing that access to helpful information and safe, accessible support is a key factor in eHealth interventions for patients with cancer [12,22]. These findings underline the importance of providing reliable and accessible information. In particular, patients benefited from the journeys and topics addressing side effects and preparation for physician consultations. These findings are further supported by our user-data analyses, which show that patients actively engaged with the app’s content. During the observation period, 81% (26/32) started a Journey module, and 50% (16/32) accessed written articles. In the follow-up period, engagement declined, but 22% (7/32) read articles, and 12% (4/32) started journeys. The frequent use of Journey modules reflects the Journey format’s strong appeal. Content analyses indicated that users were particularly interested in topics related to immunotherapy, current developments, and the management of treatment-related side effects, which was underlined by our qualitative data (ie, availability of helpful information; dealing with issues). These patterns are consistent with recent studies demonstrating that patients with cancer seek timely, therapy-specific information and guidance [25]. Future studies should take this into account when developing digital contents for patients with cancer.

However, 50% (16/32) of users did not engage with the SOFIA app’s articles. Criticisms identified in the qualitative evaluation of the SOFIA Coaching program may explain the limited use (eg, low preference for digital formats, lack of updates, or articles perceived as too general or insufficiently relevant). Future studies should provide cancer-entity-specific information, ensure regular content updates, and consider that DHIs alone may not reach all patients. To avoid disadvantaging individuals who are less digitally inclined, alternative, nondigital information formats, which are often provided by cancer-specific nongovernmental funding and nonprofit-making organizations, should continue to be offered in parallel.

### Limitations

Our study provides important insights into the assessment of ePROs and the use of DHIs in patients with cancer and represents an important addition to our feasibility study. In particular, the mixed methods approach, combining qualitative and quantitative data, represents a major strength of this study. However, several limitations have to be discussed. We only assessed the patients’ perspective in our qualitative approach. Although we quantitatively assessed physicians’ views on the benefits, usability, and use of the ePRO assessment in a sample of 33 physicians involved in treating patients in the IG [11], a complementary qualitative study would likely have provided deeper insights into perceived benefits and barriers. Due to anonymization processes, it was not possible to match the user data with other PROs and the qualitative data. Furthermore, we were unable to provide a detailed analysis of user behavior, such as engagement over time, weekly trajectories, dropout curves, and patterns of sustained vs early disengagement. Nevertheless, the data provide important insights in the usage of DHIs.

The sample for the qualitative analysis was constrained by the number of participants in the intervention, as determined by the sample size calculation for the primary outcomes of the main trial. Therefore, data saturation was not explicitly addressed in the qualitative part. However, recurring themes were identified across interviews, and our sample is larger than in comparable previous studies [12]. The concept of saturation has also been critiqued, as it assumes that no additional insights can emerge beyond a certain point [26]. Further, since the interviewers had prior contact with the participants during the recruitment phase of the study, a potential influence of social desirability has to be taken into account. However, we consider this influence to be minor, as the first contact was brief and participants also raised critical points during the phone interviews.

### Conclusions

This mixed methods study revealed the benefits and criticisms of ePRO assessments and digital coaching tools for patients with cancer under ICI-therapy. Results indicated that a user-friendly app, perceived facilitation of patient-physician communication, and several dimensions of personal value (eg, empowerment, support, or availability of helpful and interesting information) (personal values) may represent pivotal aspects for patients with cancer using DHIs. Quantitative data underline the importance of therapy-specific contents. In sum, we derived several indications for future DHIs in patients with cancer, which may contribute to the improvement of acceptance and usage and thereby further support the clinical benefit.

## Supplementary material

10.2196/91416 Multimedia Appendix 1 List of content categories of the Mika “Discover” and “Journeys” modules.

10.2196/91416 Multimedia Appendix 2 SOFIA interview guide.

10.2196/91416 Multimedia Appendix 3 Study flow chart, reproduced from Sauer et al [11], Cancer, 2024, licensed under CC BY-NC-ND 4.0.

10.2196/91416 Multimedia Appendix 4 Supplement S5 for articles read.

10.2196/91416 Multimedia Appendix 5 Supplement S6 for journeys.

10.2196/91416 Multimedia Appendix 6 Supplement S7 for exercises.

10.2196/91416 Multimedia Appendix 7 Joint display table.

10.2196/91416 Checklist 1 COREQ checklist.

## Abbrevations

COREQ: Consolidated Criteria for Reporting Qualitative Research
DHI: digital health intervention
ePROs: electronic patient-reported outcomes
HRQoL: health-related quality of life
ICI: immune checkpoint inhibitor
IG: intervention group
SOFIA: eHealth intervention for managing symptoms of immunotherapy
