# Supplementary material for: Digital Intervention for Electronic Patient-Reported Outcomes in Advanced Cancer: Mixed Methods Study
Source: JMIR Cancer. 2026 Jun 18;12:e91416. doi: 10.2196/91416 (PMC13278620; doi:10.2196/91416)
Supplement: Multimedia Appendix 1 [file cancer-v12-e91416-s001.docx]

Supplement S1

Table S1. List of content categories of the Mika ‘Discover’ and ‘Journeys’ modules

| ***Discover* module:**  **Library consisting of educational articles and videos in the following categories:** | ***Journeys* module:**  **Psycho-oncological training courses to support coping with cancer-associated distress in the following areas:** |
| --- | --- |
| Cancer and treatment types | Stress relief |
| Symptoms and side effects | Gain control |
| Nutrition in cancer | Find your way |
| Healthy lifestyle | Sources of strength |
| Partnership and family | Coping with emotions |
| Relaxation | Accept your body |
| Exercise training | Alleviate fatigue |
| Law and finances | Making decisions |
| COVID-19 and cancer | Living with immunotherapy |
| Recipes | Ovarian cancer treater |
| Survivor stories | Living with breast cancer |
|  | Yoga and cancer |
|  | Nutrition and cancer |
