# Supplementary material for: Digital Intervention for Electronic Patient-Reported Outcomes in Advanced Cancer: Mixed Methods Study
Source: JMIR Cancer. 2026 Jun 18;12:e91416. doi: 10.2196/91416 (PMC13278620; doi:10.2196/91416)
Supplement: Multimedia Appendix 2 [file cancer-v12-e91416-s002.docx]

***SOFIA Interview Guide T2 (Final Survey)***

***Version 15.04.2021***

Patient ID:_____ ____________

Interviewer:_________________

Date:___________________

End of SOFIA:_______________

How satisfied were you with the use of SOFIA overall?

Not at all satisfied Very satisfied

How helpful did you find SOFIA overall?

Not at all helpful Very helpful

1. **SOFIA Monitoring**

How satisfied were you with the application of SOFIA Monitoring?

Not at all satisfied Very satisfied

How helpful did you find SOFIA Monitoring?

Not at all helpful Very helpful

What did you find helpful about using SOFIA Monitoring?

______________________________________________________________________________________________________________________________________________________________________________________________________________________________________________________

To what extent did you use SOFIA Monitoring during your consultations with your doctor?

______________________________________________________________________________________________________________________________________________________________________________________________________________________________________________________

Did SOFIA Monitoring make the consultation with your doctor easier?

○ Yes ○ No

__________________________________________________________________________________

What did you find difficult about using SOFIA Monitoring?

______________________________________________________________________________________________________________________________________________________________________________________________________________________________________________________

What did you find lacking in SOFIA Monitoring?

______________________________________________________________________________________________________________________________________________________________________________________________________________________________________________________

1. **SOFIA COACHING**

How satisfied were you with using SOFIA Coaching?

Not at all satisfied Very satisfied

How helpful did you find SOFIA Coaching?

Not at all helpful Very helpful

What did you find helpful about using SOFIA Coaching?

______________________________________________________________________________________________________________________________________________________________________________________________________________________________________________________

What did you find difficult about using SOFIA Coaching?

______________________________________________________________________________________________________________________________________________________________________________________________________________________________________________________

What did you find lacking in SOFIA Coaching?

______________________________________________________________________________________________________________________________________________________________________________________________________________________________________________________

Any other comments?

______________________________________________________________________________________________________________________________________________________________________________________________________________________________________________________
