# Supplementary figures and images for: Digital Intervention for Electronic Patient-Reported Outcomes in Advanced Cancer: Mixed Methods Study
Source: JMIR Cancer. 2026 Jun 18;12:e91416. doi: 10.2196/91416 (PMC13278620; doi:10.2196/91416)

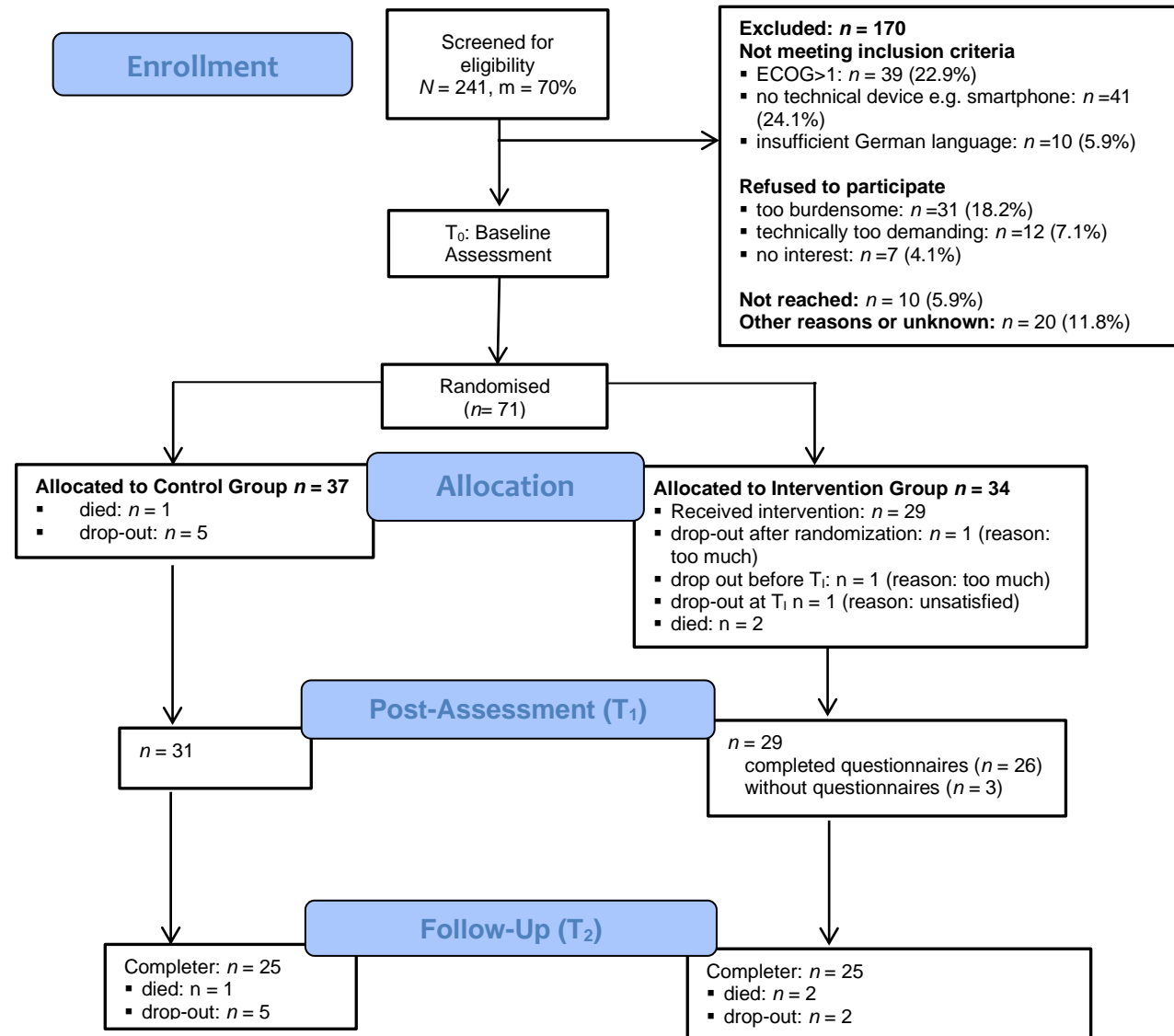

Supplement: Multimedia Appendix 3 [file cancer-v12-e91416-s003.pdf]

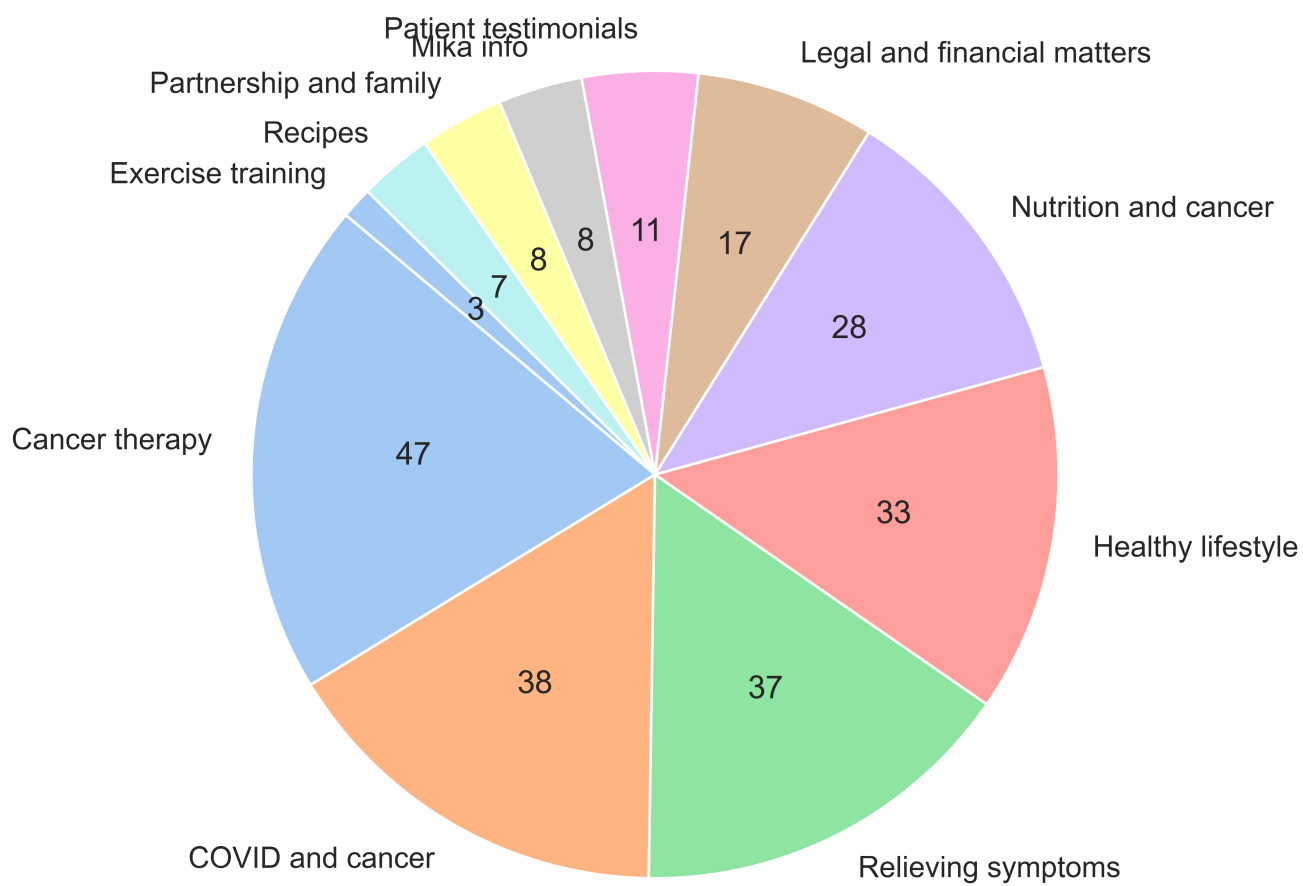

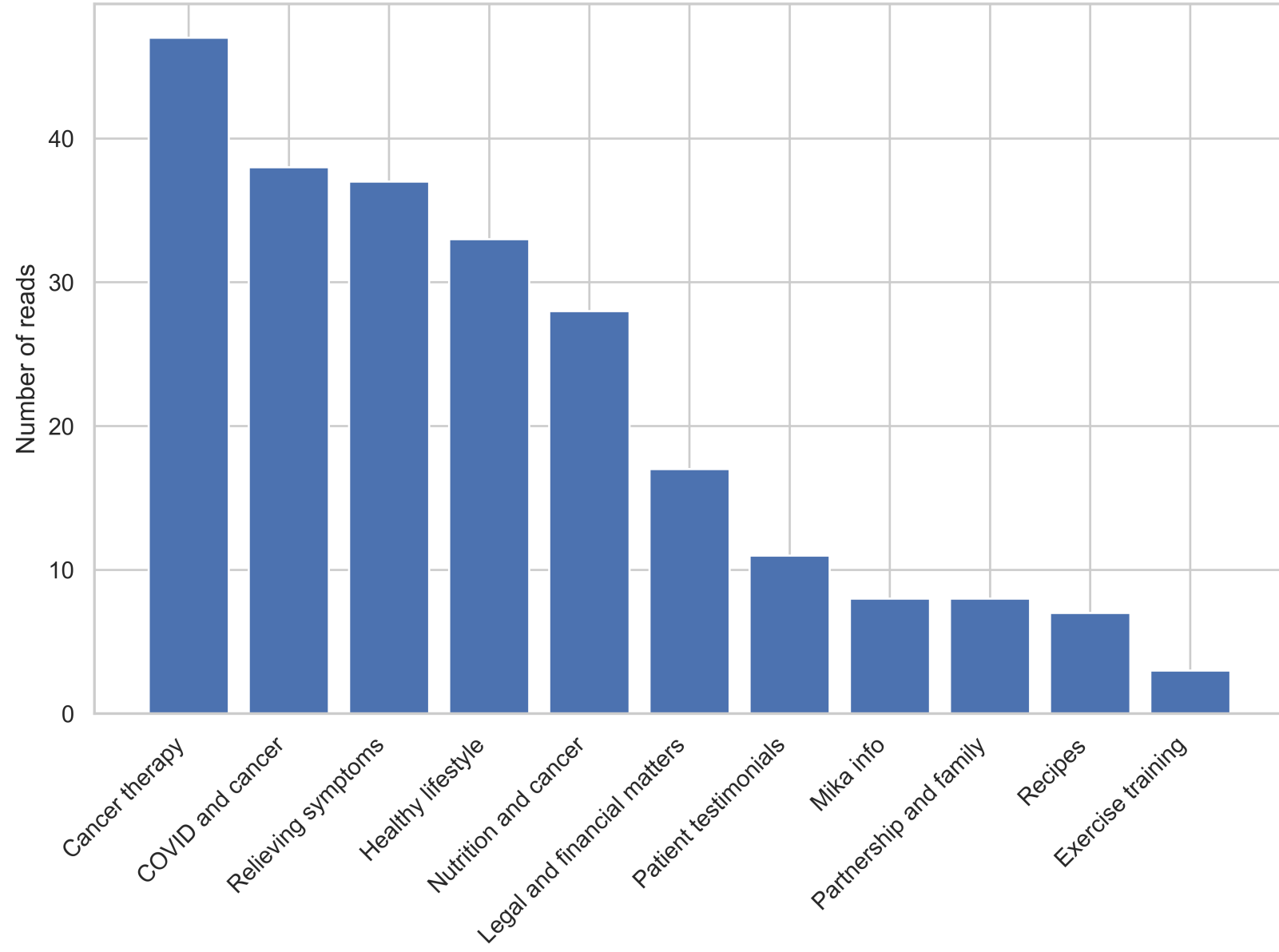

Number of articles read per user (both IV and FU periods combined)

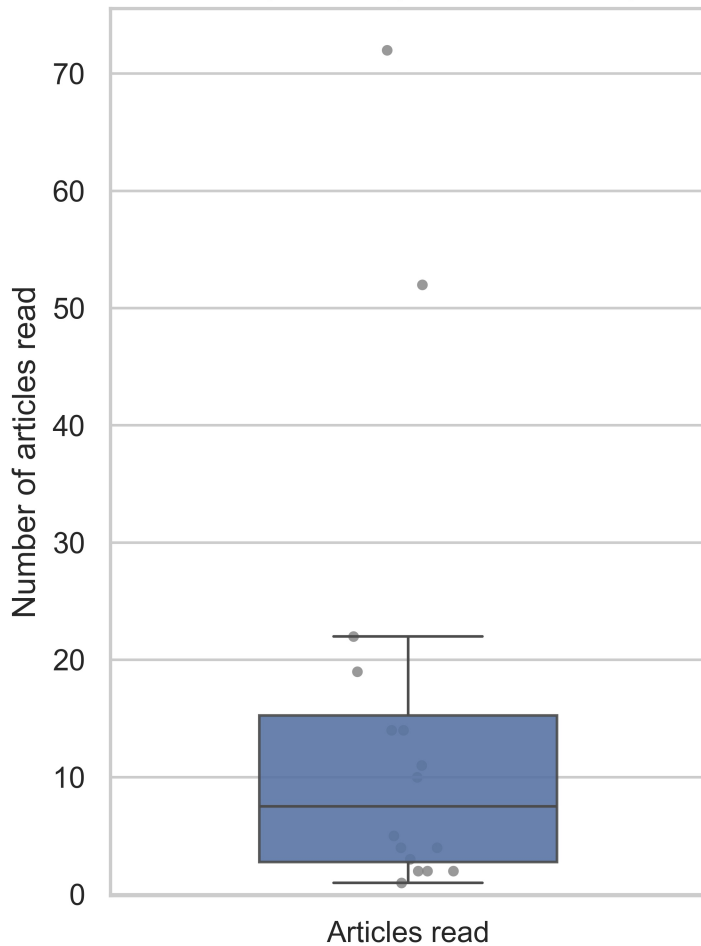

Supplement: Multimedia Appendix 4 [file cancer-v12-e91416-s004.pdf]

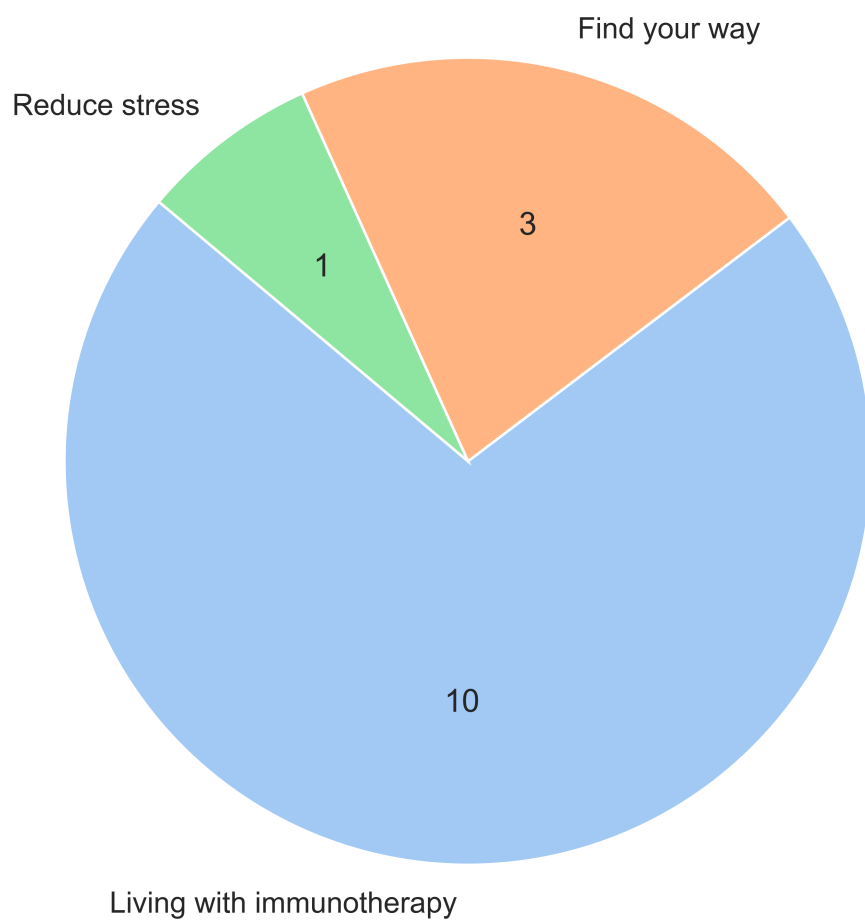

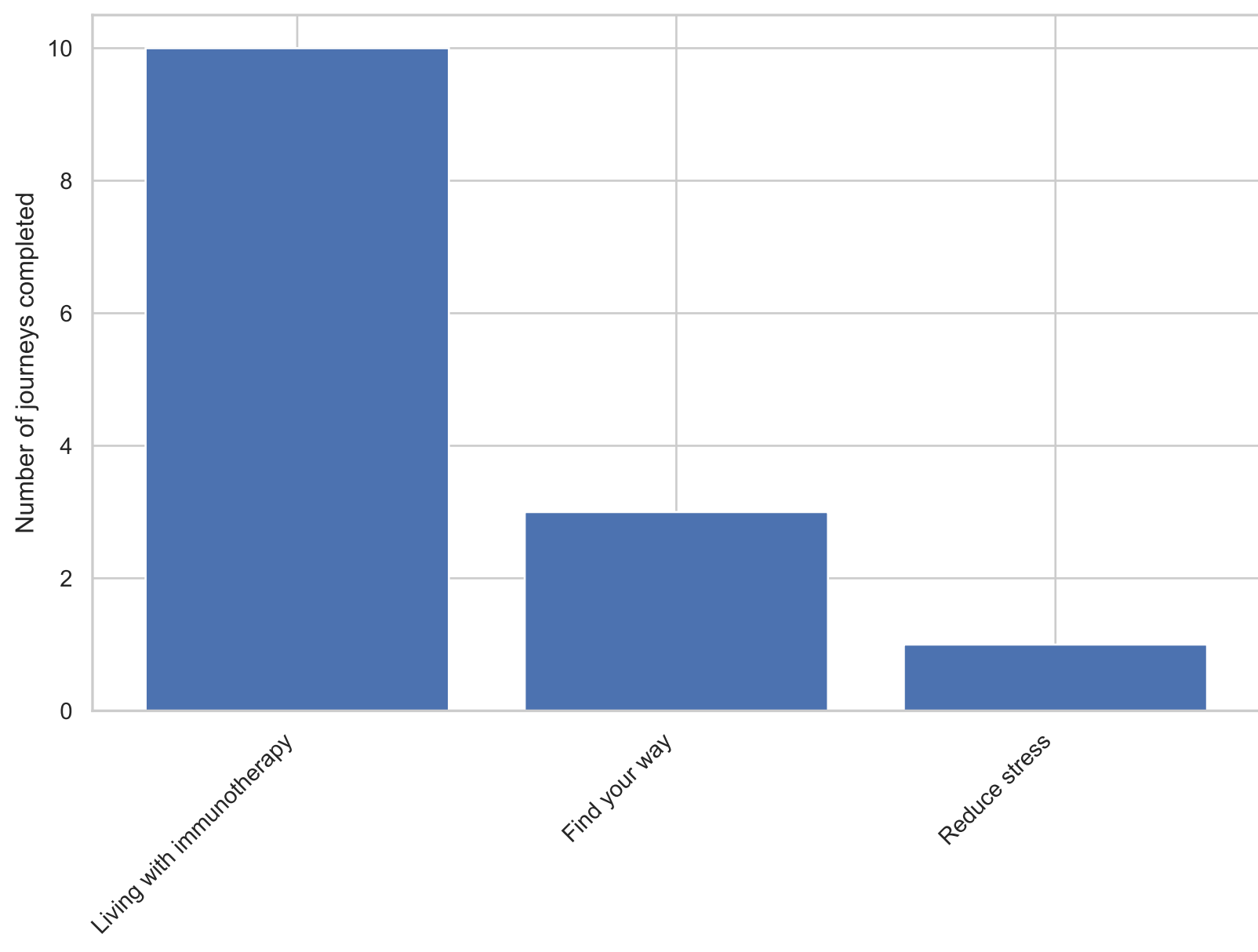

Number of journeys started and completed per user (both IV and FU periods combined)

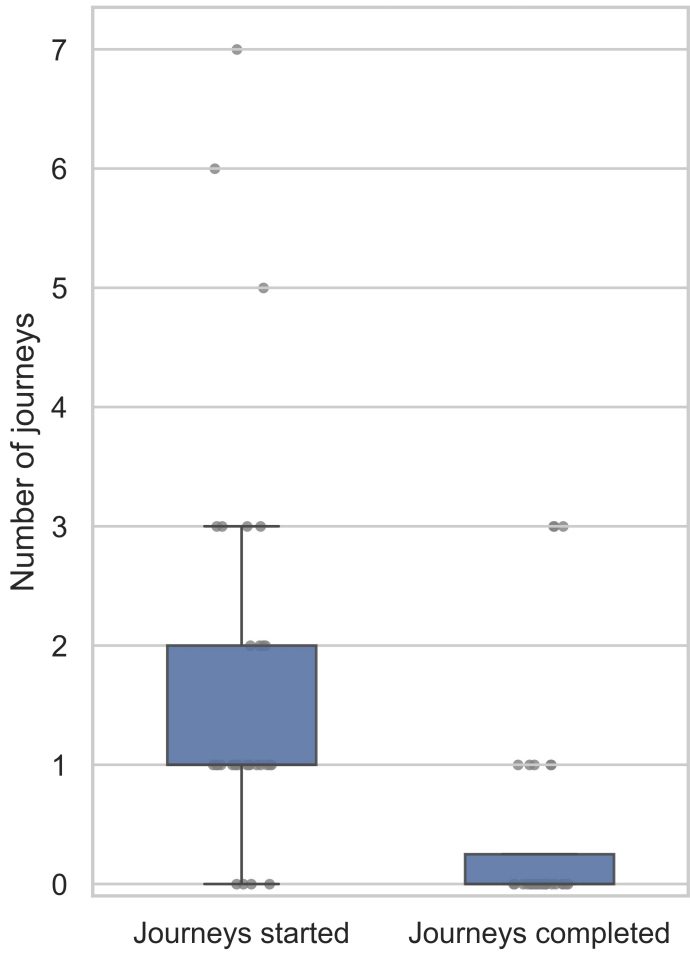

Supplement: Multimedia Appendix 5 [file cancer-v12-e91416-s005.pdf]

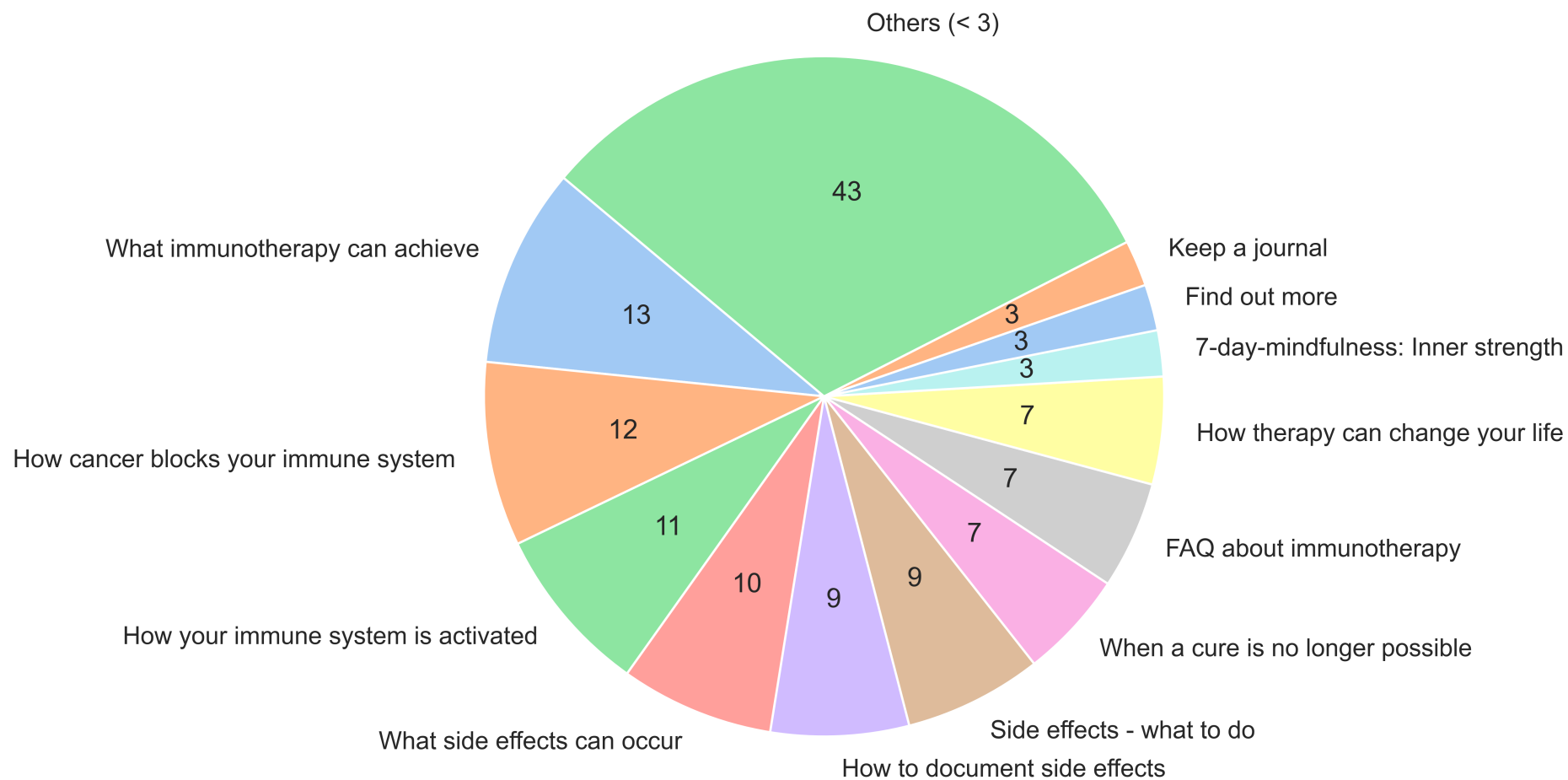

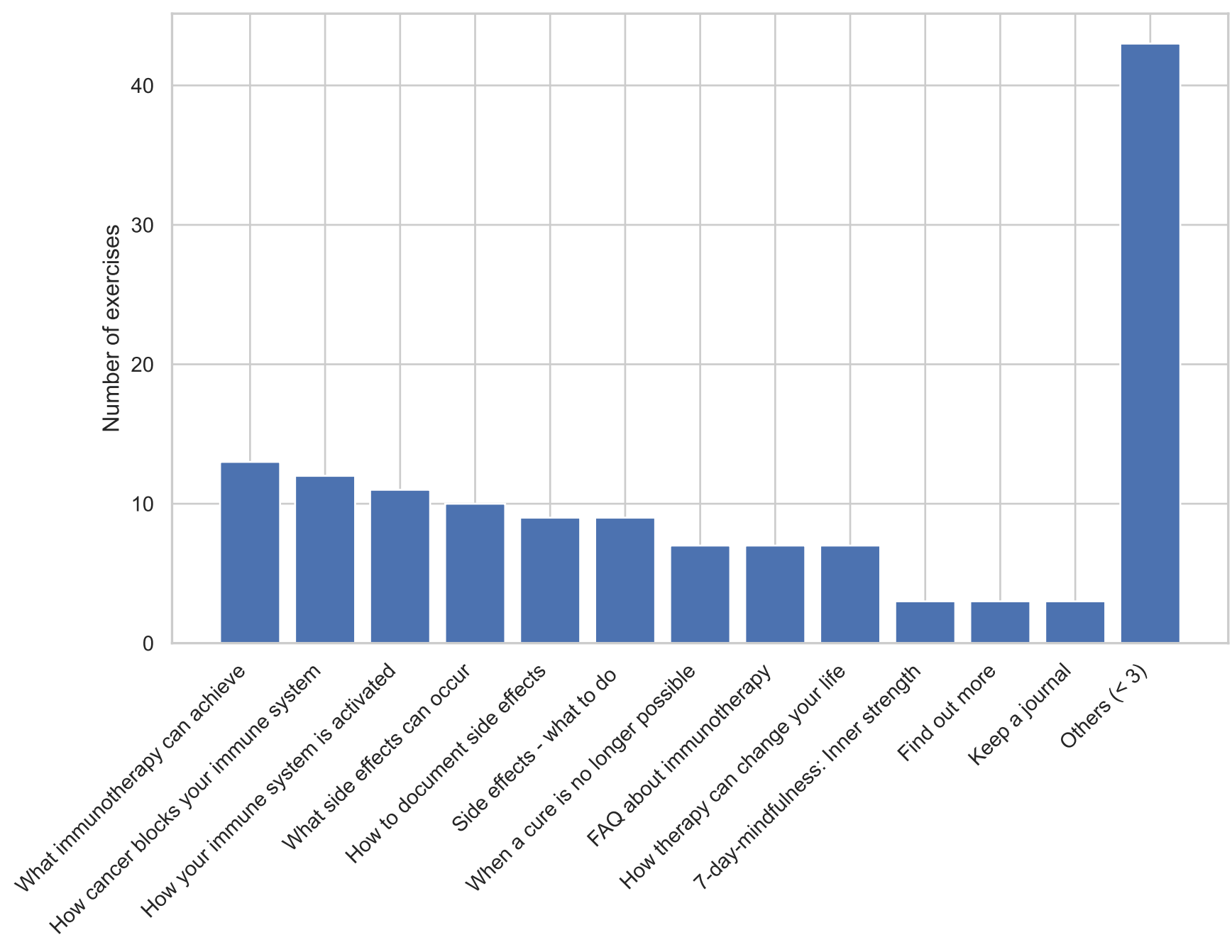

Supplement: Multimedia Appendix 6 [file cancer-v12-e91416-s006.pdf]
