# Supplementary material for: Digital Intervention for Electronic Patient-Reported Outcomes in Advanced Cancer: Mixed Methods Study
Source: JMIR Cancer. 2026 Jun 18;12:e91416. doi: 10.2196/91416 (PMC13278620; doi:10.2196/91416)
Supplement: Multimedia Appendix 7 [file cancer-v12-e91416-s007.docx]

S8: Joint Display Table Sauer et al.

| **SOFIA Coaching Benefits** | | |
| --- | --- | --- |
| **Generic Category: Availability of helpful information** | | |
| **Subcategory/ Qualitative Finding** | **Quantitative Finding** | **Integrated Interpretation** |
| Helpful and interesting information  *“One can glean useful tips from it.”* | - 16 users (50%) have read at least one article from the 'Discover' module. - Median number of articles read per user was 7.5. - 28 users (87.5%) started at least one journey; 8 of them (25%) completed at least one journey. - 15 users (46.9%) were engaged in an exercise | - Both quantitative and qualitative findings converge in highlighting the importance of providing reliable and accessible information. |
| Specific modules used  *“Thematic journeys, approximately every two days for half an hour to an hour, are helpful.”* | - 28 users (87.5%) started at least one journey; 8 of them (25%) completed at least one journey. - most frequently started journeys: “Living with immunotherapy” (*k* = 24), “Find your way” (*k* = 8), “Relieve exhaustion” *(k* = 6), “gain control” (*k* = 5) - frequently read categories: “cancer therapy” (*k* = 47), "COVID and cancer” (*k* = 38), "relieving symptoms” (*k* = 37), "healthy lifestyle” (*k* = 33), "nutrition and cancer” (*k* = 28) | - Convergence across datasets regarding the importance of thematic journeys and cancer- and immunotherapy-related topics. |
| Addressing issues  *“It's good that it's included in the app, because otherwise you might not bother with it. Depending on what's happening that day, you can take another look at it.”* | - frequently read categories: “cancer therapy” (*k* = 47), "COVID and cancer” (*k* = 38), "relieving symptoms” (*k* = 37), "healthy lifestyle” (*k* = 33), "nutrition and cancer” (*k* = 28) - most frequently started journey: “Living with immunotherapy” (*k* = 24) | - Convergence regarding interest in immunotherapy and side-effect management as well as treatment-relevant information. |
| Encourages patient-physician communication  *“I looked at thematic journeys, which raised questions that I then asked during my consultation with the doctor.”* | - frequently read categories: “cancer therapy” (*k* = 47), "COVID and cancer” (*k* = 38), "relieving symptoms” (*k* = 37), "healthy lifestyle” (*k* = 33), "nutrition and cancer” (*k* = 28) - 28 users (87.5%) started at least one journey - most frequently started journeys: “Living with immunotherapy” (*k* = 24), “Find your way” (*k* = 8), “Relieve exhaustion” (*k* = 6), “gain control” (*k* = 5) | - Both datasets converge in showing highest interest in immunotherapy and the management of treatment-related side effects, which were also used to support patient-physician communication. |
